# Induction Systemic Therapy Followed by Response-Adapted Consolidative Radiation in Locally Advanced Non-Melanoma Skin Cancer: A Retrospective Case Series

**DOI:** 10.3390/curroncol33100602

**Published:** 2026-10-06

**Authors:** Poorva Vaidya, Tali Azenkot, John Bliamptis, Mitchell Toby Victor, Anna Dornisch, Jennifer Chang, Parag Sanghvi, Theresa Guo, Soo J. Park

**Affiliations:** 1 Chao Family Comprehensive Cancer Center, University of California, Orange, CA 92868, USA; poorvav@hs.uci.edu; 2 Division of Medical Oncology, Moores Cancer Center, University of California, San Diego, CA 92037, USA; 3 Department of Otolaryngology-Head and Neck Surgery, Indiana University Health, Indianopolis, IN 46202, USA; 4 Department of Radiation Medicine and Applied Sciences, University of California, San Diego, CA 92037, USA; 5 Department of Radiology, University of California, San Diego, CA 92037, USA; 6 Department of Otolaryngology-Head and Neck Surgery, University of California, San Diego, CA 92037, USA

**Keywords:** cutaneous squamous cell carcinoma, basal cell carcinoma, Merkel cell carcinoma, immunotherapy, radiation, neoadjuvant

## Abstract

Background: There exists an unmet clinical need to define definitive management strategies for patients with locally advanced non-melanoma skin cancers (NMSC), particularly if not amenable to surgical resection. Methods: We retrospectively reviewed a case series of locally advanced unresectable NMSC treated with induction systemic treatment followed by response-adapted consolidative radiation. Results: We identified 15 patients who met study criteria, including seven with basal cell carcinoma (BCC), six with cutaneous squamous cell carcinoma (CSCC), and two with Merkel cell carcinoma (MCC). Clinical complete response (CR) by physical examination was observed in all 15 patients. No locoregional or distant recurrences were observed in any histologic subtype after completion of radiation (median follow-up from completion of radiation: 18.1 months BCC, 22.5 months CSCC, 22.7 months MCC). The median duration of observed response (DOOR), measured from first clinical response was 26.9 months (26.2 BCC, 29.4 CSCC, 27.1 MCC). Radiation treatment volumes were response-adapted with median gross tumor volume reduction of 55.3% (15.3%–100%). Median treatment-free response was 20.5 months. No unexpected systemic therapy related toxicities were identified. Conclusions: In this single-institution retrospective case series, induction systemic therapy followed by response-adapted consolidative radiation was feasible and associated with durable disease control in selected patients with locally advanced unresectable NMSC.

## Simple Summary

Non-melanoma skin cancers (NMSC) include cutaneous squamous cell carcinoma, basal cell carcinoma, and Merkel cell carcinoma. While early-stage disease can be treated with upfront excision, locally advanced disease poses a greater therapeutic challenge as surgery can cause significant cosmetic morbidity, tumors tend to occur in an elderly population at high risk for surgery, and many advanced tumors are at high risk of recurrence. Here, we present a retrospective case review of patients with locally advanced NMSC not amenable to upfront resection, treated with induction systemic therapy followed by response-adapted consolidative radiation.

## 1. Introduction

Standard treatment for locally advanced non-melanoma skin cancer (NMSC) is surgical excision, followed by consideration of adjuvant radiation and immunotherapy if high-risk features are present. These patients may require large resections in cosmetically sensitive areas and often have poor outcomes, including locoregional recurrence, metastasis, and death [1]. For some patients, tumors are not amenable to upfront resection due to surgical morbidity or patient comorbidities, especially in the elderly population for which NMSC incidence is rising [2,3]. Many of these cases are considered incurable with local therapies. Recently, the use of systemic therapy early in the treatment paradigm has opened the door for alternatives to surgery. 

Until the development of immune checkpoint inhibitors (ICI) and hedgehog inhibitors (HHI), systemic therapy options for advanced NMSC were limited. In the locally advanced and metastatic settings, sonidegib and vismodegib are HHIs approved for treatment of advanced basal cell carcinoma (BCC), while PD-1 inhibitors are approved for cutaneous squamous cell carcinoma (CSCC), and PD-1 (pembrolizumab, retifanlimab) and PD-L1 (avelumab) inhibitors are approved for Merkel cell carcinoma (MCC) [4,5,6,7,8]. More recently, ICIs have shown significant promise in the neoadjuvant setting with the aim of downstaging tumors prior to resection and hypothetically priming immunity to potentially establish a more durable immune response [9,10,11,12,13]. For CSCC, the largest trial to date was a phase II trial which showed a 51% complete pathologic response (pCR) and 13% major pathologic response to neoadjuvant cemiplimab [10,14]. Post hoc exploratory analyses from patients receiving cemiplimab for advanced CSCC indicate that patients who received this treatment approach reported improved health-related quality of life outcomes [15]. In MCC, neoadjuvant nivolumab achieved a 47% pCR [16]. While clinical trials for ICIs in the neoadjuvant setting for BCC are currently ongoing [17], neoadjuvant vismodegib was reported to have a 57% complete response based on biopsy rather than surgical pathology [18]. However, the 3-year recurrence rate was high at 36% with an additional 27% lost to follow-up, suggesting that adjuvant therapy or alternative neoadjuvant strategies are necessary [18].

In the context of a paradigm shift toward neoadjuvant treatment for NMSC, an induction systemic therapy approach followed by consolidative radiation may also yield durable responses. While prior reports have suggested that this combination of treatment is effective, investigation of this approach has been limited [19]. In this retrospective case series, we describe our experience with induction systemic therapy followed by response-adapted consolidative radiation as a treatment approach for patients with locally advanced unresectable NMSC.

## 2. Materials and Methods

This study was approved by the University of California San Diego Institutional Review Board, protocol ID 806937. The study included patients diagnosed with unresectable NMSC who were treated with induction systemic therapy in combination with radiation at Moores Cancer Center. Medical chart abstraction was conducted to gather data on demographics, tumor size and location, stage, the reason surgery was not pursued, and treatment details for systemic therapy and radiation. 

All patients were discussed at a multidisciplinary tumor board with multiple surgeons, medical oncologists, dermatologists, pathologists, and radiologists. Reasons for not pursuing surgery included technically unresectable disease (such as critical vascular encasement or intracranial or skull base involvement), surgical co-morbidities (which would deem resection medically high risk) or patients declining surgery (including for cosmetic reasons). All treatment approaches were thoroughly reviewed with each patient and shared decision making was respected. After consensus regarding inoperability, patients started systemic treatment, which included sonidegib 200 mg PO daily for BCC, cemiplimab 350 mg IV every three weeks for CSCC, and pembrolizumab 200 mg IV every 3 weeks for MCC. Patients with contraindications or comorbidities precluding safe administration of HHI or ICI were not treated with this paradigm. Treatment details including type and duration of systemic therapy, timing of radiation, and radiation dose were obtained. The gross tumor volume (GTV) was contoured on both pre-treatment imaging and the radiation simulation scan using MIM (Version 7.2.6, MIM Software Inc.; Beachwood, OH, USA). Volume (in cc) of GTVs was calculated and percent change in GTV was measured to determine GTV reduction. If there was no visible tumor on the simulation scan, this was calculated as 100% GTV reduction. For post-systemic radiation treatment, the initial extent of disease with account for appropriate contraction from normal anatomy was covered. The post-systemic therapy gross disease was treated to the ablative dose (60–66 Gy extracranial based on normal structure tolerance); however, the pre-systemic treatment areas that had resolved were treated to a microscopic dose of 50–54 Gy. For MCC, circulating tumor DNA (ctDNA) levels were monitored at baseline and after each treatment cycle using the Signatera^TM^ molecular residual disease assay (Natera; Austin, TX, USA) [20]. This method was used for MCC and not the other histological subtypes because circulating tumor DNA (ctDNA) has been validated as a reliable biomarker for MCC disease activity and is predictive of recurrence [20]. Radiation was initiated after ctDNA reached undetectable levels on at least two consecutive draws to confirm minimal residual disease, an approach which remains exploratory and requires prospective data for validation. Data regarding adverse drug reactions were collected and graded by Common Terminology Criteria for Adverse Events Version 5.0 (CTCAE v5). Subsequent dose reduction or cessation of the systemic agent was documented. 

Clinical response to treatment was measured retrospectively by reviewing documentation of the physical examination by the medical oncologist during clinical visits and by review of post-treatment imaging by a board-certified neuroradiologist using the Response Evaluation Criteria in Solid Tumors version 1.1 (RECIST v1.1). Imaging was obtained after completion of radiation, except in the case of one patient with auricular BCC, whose post-radiation imaging did not fully include the primary disease site. Clinical photographs were obtained at medical oncology visits: pre-treatment, during treatment, and post-treatment. Clinical complete response (CR) by physical exam was defined as lack of visible and palpable tumor, healing of cutaneous ulcerations, and full skin epithelization as determined by both physical examination and photo-documentation. Duration of observed response (DOOR) was defined as the time from first clinical response by physical examination meeting the above criteria to the last oncology-related encounter. Because first response occurred during induction systemic therapy, DOOR includes time on treatment and reflects the period over which response was observed rather than a conventional duration of response. The treatment-free interval was defined as the time from completion of all treatment (systemic therapy or radiation, whichever ended later) to the last oncology-related encounter. Follow-up after treatment was also measured from the last day of radiation and is reported separately for each histologic subtype. Given the small number of patients within each subtype and the absence of recurrence events, time-to-event (Kaplan–Meier) estimates were not considered informative and are not reported. Because Patients 8 and 12 did not follow the induction-then-consolidation sequence, outcomes were also reported in a sensitivity analysis excluding these two patients. Patients with MCC continue to have ctDNA monitored every 3 months. 

## 3. Results

### 3.1. Baseline Characteristics

Fifteen patients with pathologically confirmed BCC, CSCC, or MCC who were not candidates for surgical resection and were candidates for systemic treatment were identified (Table 1). Seven patients (47%) were diagnosed with BCC, six (40%) with CSCC, and two (13%) with MCC. Median age at diagnosis was 72 years for BCC, 74 years for CSCC, and 62 years for MCC. Thirteen (87%) were male and two (13%) were female. Tumors were located on the face (6, 40%), ear (4, 27%), scalp (2, 13%), upper extremity (2, 13%), and neck (1, 7%). Surgery was deferred due to tumor size in 10 (67%) patients, cosmetic morbidity in three (20%) patients, and dermal lymphatic spread in two (13%) patients. AJCC 8th edition stage, index lesion size, nodal status, and perineural invasion for each patient are provided in Supplementary Table S1. Median follow-up was 28.2 months from the start of systemic therapy. Measured from completion of radiation, median follow-up was 18.1 months (range 3.1–23.5) for BCC, 22.5 months (range 3.4–55.3) for CSCC, and 22.7 months (range 16.7–28.8) for MCC. 

### 3.2. Treatment

**Figure 1:**
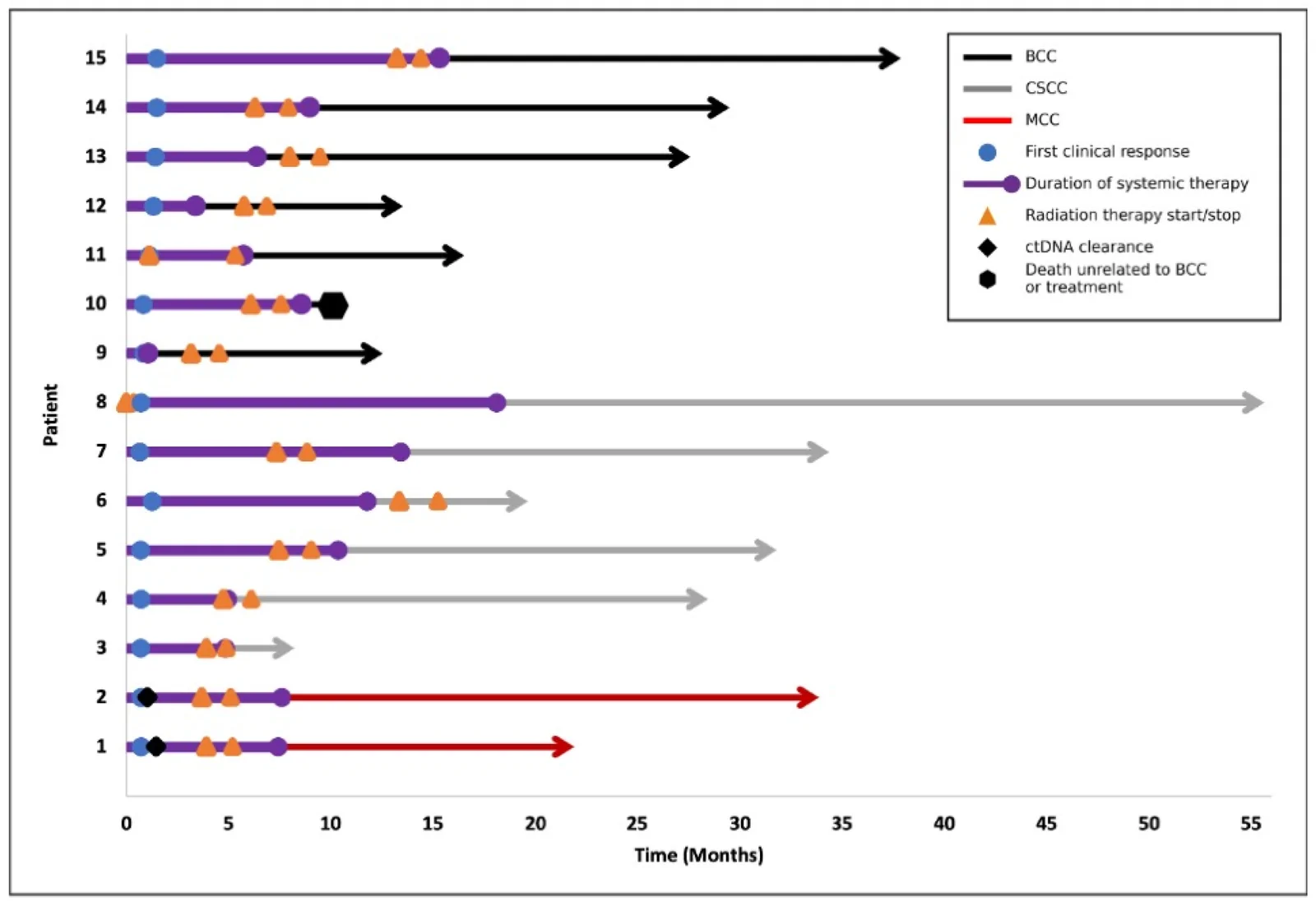
Duration of observed clinical response for 15 patients from start of systemic therapy (Swimmer’s plot). Histologic subtypes are depicted by color: BCC (black), CSCC (gray), and MCC (red). First clinical response is marked by blue dots; duration of systemic therapy is marked in purple; radiation therapy start and stop dates are marked by orange triangles; ctDNA clearance time is marked for Patients 1 and 2 with MCC by black diamonds; and death of Patient 10 from causes unrelated to BCC or treatment is marked by the black hexagon. Note Patient 12 initially started definitive radiation but stopped due to intolerance; systemic therapy was started with excellent cytoreduction and then response-adapted consolidative radiation was delivered. Patient 8 started definitive radiation for CSCC, but then shortly after developed in-transit dermal metastases requiring concurrent systemic immunotherapy and radiation. Abbreviations: BCC = basal cell carcinoma; CSCC = cutaneous squamous cell carcinoma; MCC = Merkel cell carcinoma.

All patients with BCC were treated with sonidegib with median duration of treatment of 6.4 months (Table 1). Patients with CSCC were all treated with cemiplimab with a median duration of treatment of 11.1 months. Two patients with MCC were treated with pembrolizumab with median treatment duration of 7.5 months. Systemic therapy duration varied widely (1.1–18.8 months) because it was individualized: therapy was continued until maximal clinical cytoreduction as judged by the treating medical oncologist, was shortened by toxicity (e.g., Grade 3 hepatitis in Patient 4), and in nine patients was continued during or after radiation. Radiation therapy was initiated after obtaining maximal clinical cytoreduction to systemic therapy per determination by medical oncology with a median treatment time of 1.4 months. For the two patients with MCC, previously detectable ctDNA levels were negative on two sequential checks prior to start of radiation. Treatment timelines, clinical responses, and ctDNA clearance are shown in Figure 1. Patient 8, with CSCC, did not follow the standard protocol as they started definitive RT but then developed in-transit dermal metastases requiring concurrent systemic immunotherapy and radiation. Pre-treatment baseline Natera ctDNA values were 40.78 MTM/mL for Patient 1 and 60.18 MTM/mL for Patient 2.

All patients were treated with consolidative radiation therapy with external beam radiation therapy (EBRT) with high energy photons or electrons delivered by a linear accelerator. Patients with CSCC were treated to 60–70 Gy in 30–35 fractions with conventional fractionation with Intensity Modulated Radiation Therapy (IMRT) with photons; elective lymph node basins were covered for two patients with CSCC due to known nodal disease and primary ear location at high risk of occult nodal failure. Patients with BCC were treated to 55–66 Gy in 20–33 fractions, either with photon IMRT or electrons at the discretion of the radiation oncologist; elective node basins were covered in only one patient due to radiographic invasion of the level 2 nodal stations. Patients with MCC were treated to 56–60 Gy in 28–30 fractions with IMRT with elective lymph node coverage. 

### 3.3. Response-Adapted Radiation with Volume Reduction

**Figure 2:**
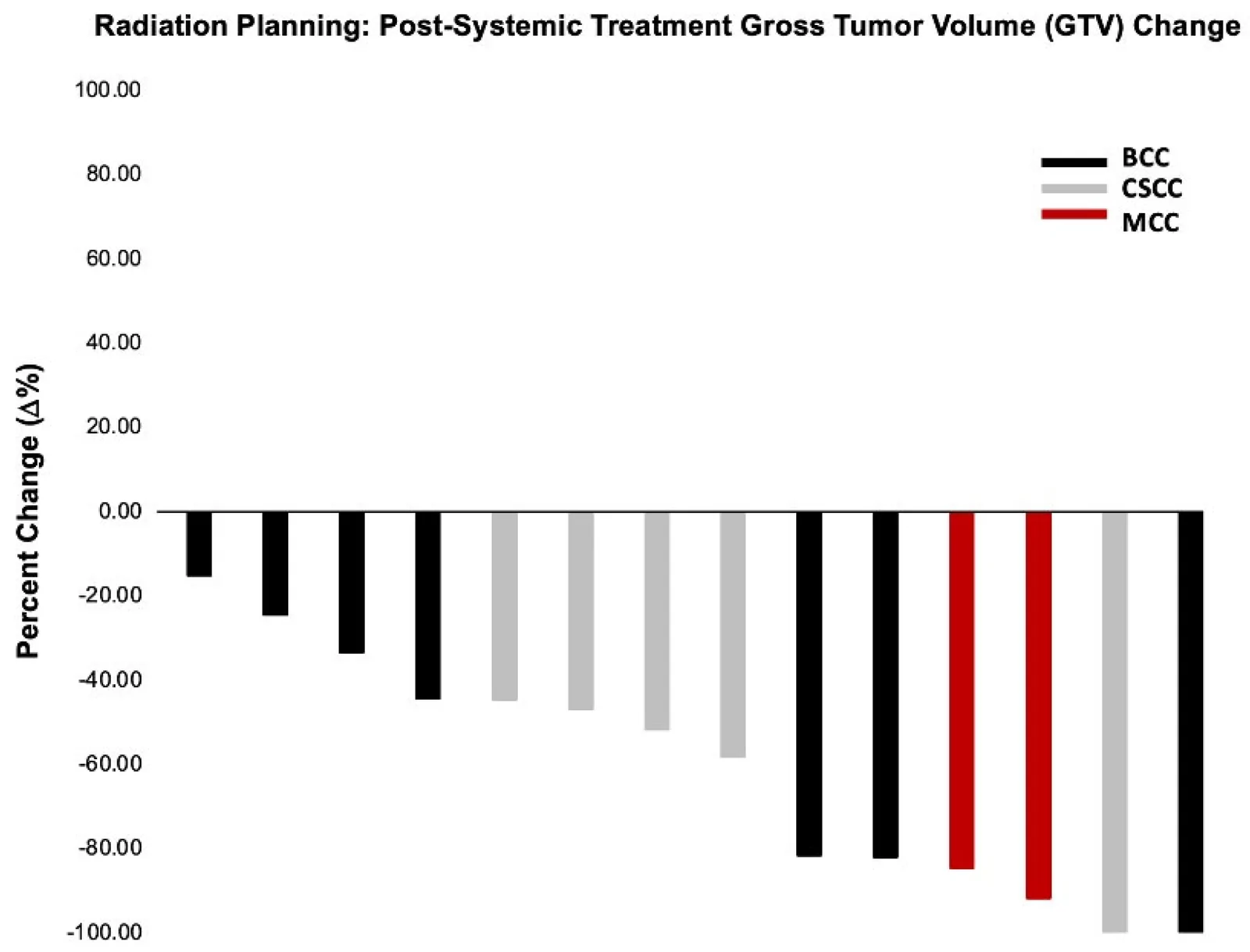
Radiation mapping indicating gross tumor irradiation change after induction systemic treatment (Waterfall plot). GTV was not mapped in one patient with CSCC as they developed in-transit dermal metastases during upfront definitive radiation and then received systemic therapy followed by concurrent radiation.

**Figure 3:**
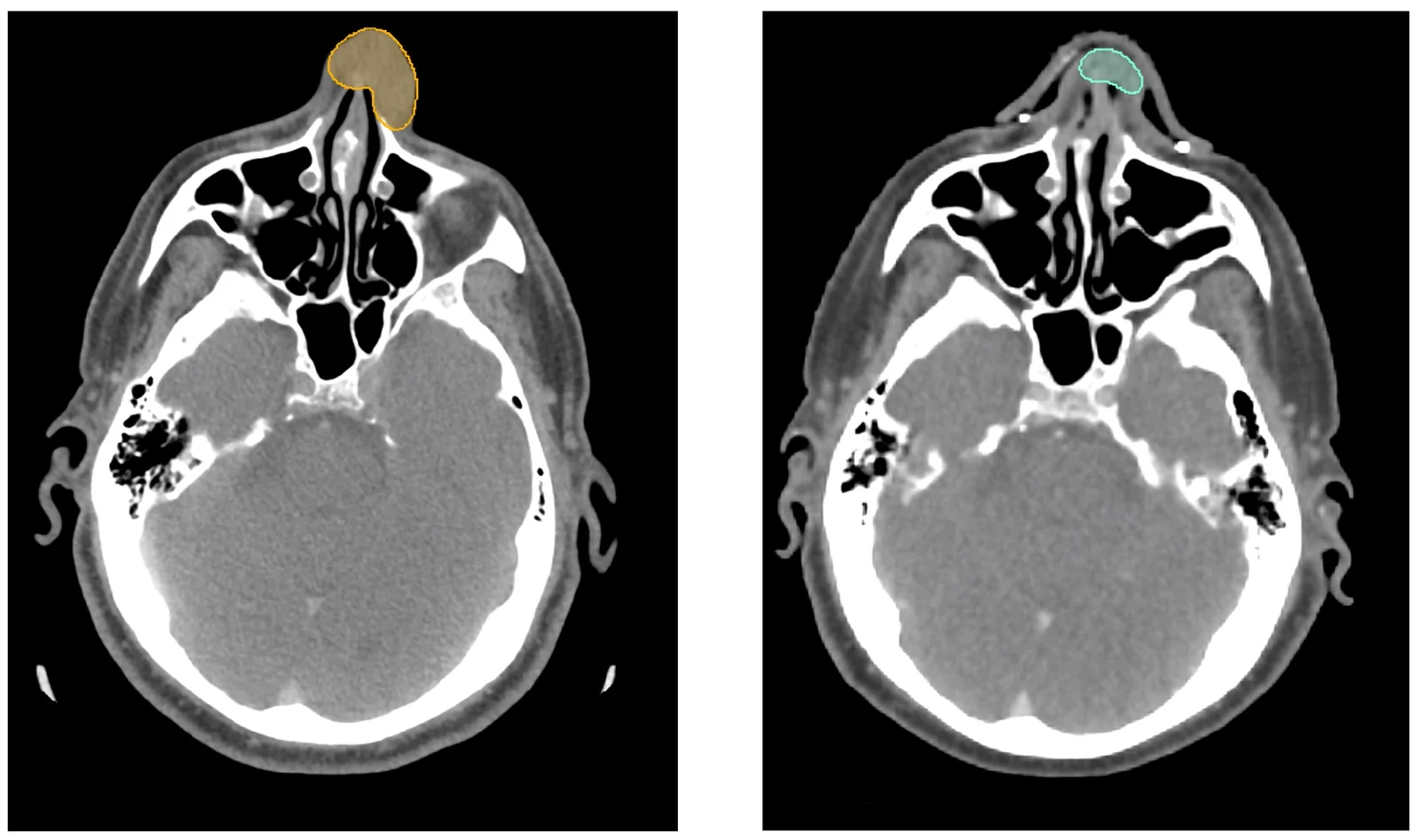
Radiation mapping (highlighted) pre- and post-systemic treatment for a patient with BCC of the nose.

All patients underwent tumor cytoreduction with systemic therapy, leading to a decrease in radiation treatment volume delivery, as calculated by the percent difference in total gross disease between the pre-treatment imaging and the radiation simulation scan (Figure 2). The median irradiated volume percent reduction was 55.27%, with a range of 15.25% to 100%. Patient 12 did not initially complete definitive radiation due to intolerance secondary to a large gross tumor volume (GTV); the patient started sonidegib 11 days later with excellent cytoreduction, followed by response-adapted consolidative radiation with an irradiated volume percent reduction of 45%. Change in GTV could not be calculated for Patient 8, the other noted protocol deviation, who started definitive radiation for CSCC, but then shortly after developed in-transit dermal metastases requiring concurrent systemic immunotherapy and radiation. An example of pre-systemic and post-systemic treatment changes in radiation mapping is exhibited in Figure 3.

### 3.4. Clinical and Radiographic Response

**Figure 4:**
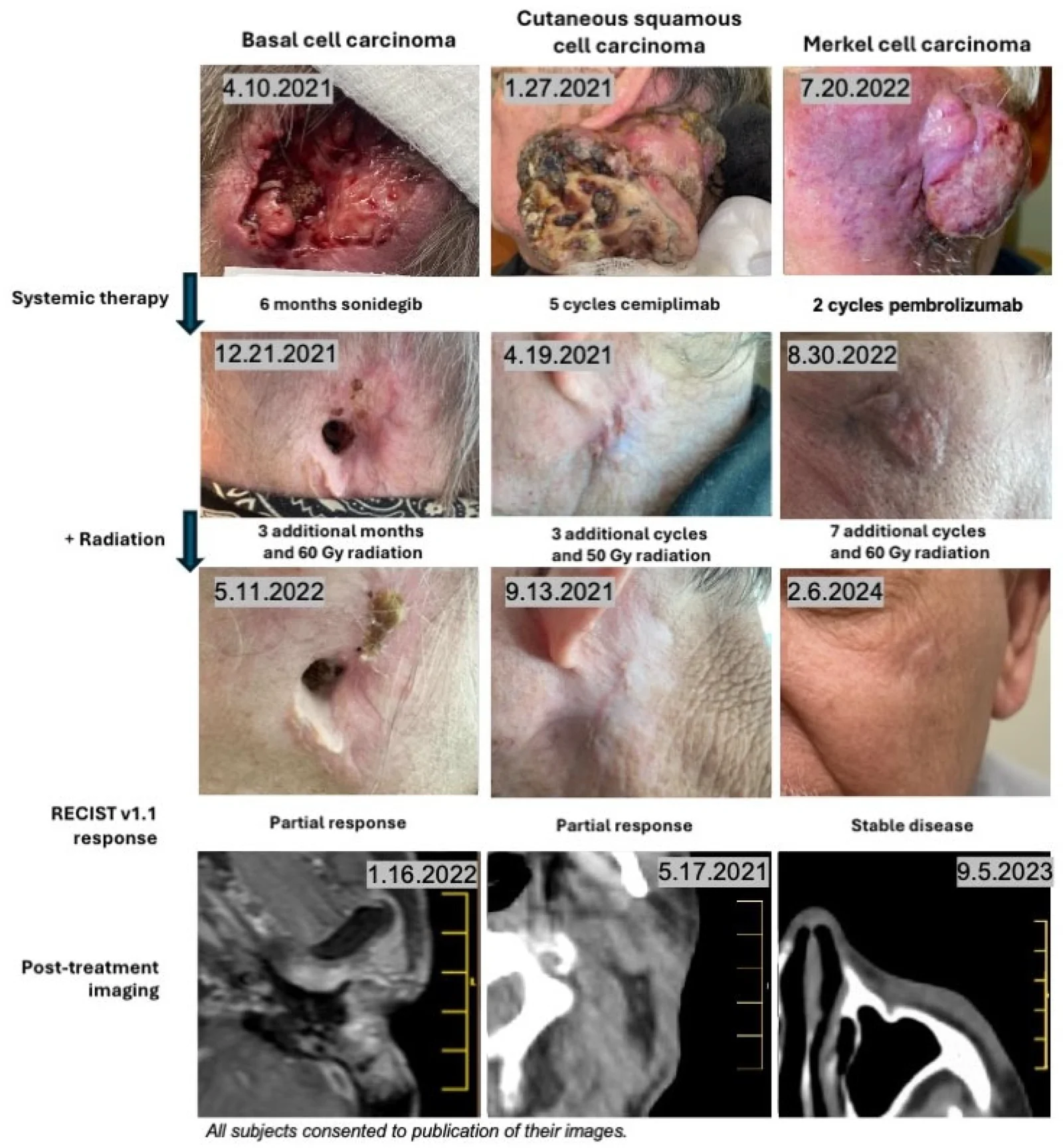
Clinical and radiographic response in select patients. Images of advanced NMSC pre-treatment (top row), following induction systemic therapy alone (second row), after completing both systemic therapy and radiation (third row), and by post-treatment CT imaging (bottom row). CR was noted on clinical exam in all three patients. Clinical and radiographic responses were not concordant: radiographic imaging shows partial response by RECIST v 1.1 in the patients with BCC and CSCC and stable disease in the patient with MCC. Scale bars are 1 cm per small box and 5 cm total. Abbreviations: BCC = basal cell carcinoma; CSCC = cutaneous squamous cell carcinoma; MCC = Merkel cell carcinoma.

A 100% clinical CR by physical exam was observed in each group. (Table 1). Median time to first clinical response was 40 days for BCC, 21.5 days for CSCC, and 22 days for MCC. Radiographic response per RECIST v1.1 was variable (Table 1). In the BCC group, three patients (43%) had a radiographic CR, one (14%) had a partial response (PR), two (29%) had stable disease (SD), and post-radiation imaging did not fully address the site of interest for one patient (14%). In the CSCC group, two patients (33%) had a CR, two (33%) had a PR, and two (33%) had SD. Examples of clinical and radiographic response for a CSCC tumor and for each NMSC histology are provided in Figure 4. In the MCC group, both patients had SD on imaging; however, ctDNA levels were detectable at the start of systemic therapy and undetectable after 44 days in one patient and 31 days in the other (mean time to ctDNA clearance was 37.5 days). 

Patients were followed for a median duration of 27.6 months for BCC, 30.1 months for CSCC, and 27.9 months for MCC, with all surviving patients exhibiting durable responses at time of data cutoff (Figure 1). No local or distant recurrences were observed following completion of radiation therapy for the entire duration of follow-up. No locoregional or distant recurrences occurred in any histologic subtype after completion of radiation. The only death was that of Patient 10 (BCC) from unrelated comorbidities 3.1 months after radiation. Four of seven patients with BCC, four of six with CSCC, and both patients with MCC were followed for at least 12 months after radiation; two patients with CSCC had less than 6 months of post-radiation follow-up (Patients 3 and 6—relocation and loss to follow-up, respectively). Median DOOR was 26.2 months for BCC, 29.4 months for CSCC, and 27.1 months for MCC. Because DOOR includes time on induction therapy, it should be interpreted as the duration over which response was observed rather than as a conventional duration of response. The longest duration of observed response from the first clinical response was 36.4 months for BCC, 54.9 months for CSCC, and 33.1 months for MCC. Median treatment-free response was 18.1 months for BCC, 21.2 months for CSCC, and 20.4 months for MCC. One patient with a right mandibular cheek CSCC started definitive radiation but rapidly developed in-transit dermal metastases after receiving less than 20 Gy of radiation (Patient 8, Figure 1). Cemiplimab was added concurrently prior to receiving therapeutic doses of radiation. This patient then completed concurrent ICI and radiation and continues to have a durable response at 54.9 months. In a sensitivity analysis excluding the two patients whose treatment deviated from the induction-then-consolidation sequence (Patients 8 and 12), median DOOR was 20.8 months for BCC (n = 6) and 27.7 months for CSCC (n = 5), and the median treatment-free interval was 14.4 months for BCC and 20.9 months for CSCC; MCC results were unchanged. No locoregional or distant recurrences occurred in either subgroup. The two patients with MCC continue to have undetectable ctDNA levels at 32.4 and 20.3 months after initial clearance. 

### 3.5. Adverse Events

Adverse events occurred in four patients with BCC on sonidegib and one patient with CSCC on cemiplimab. Four patients (57%) on sonidegib experienced Grade 2 dysgeusia with two requiring dose reduction at every other day due to poor oral intake and failure to thrive. One of these patients (14%) in the BCC group also experienced Grade 2 nausea leading to dose reduction. Of the six patients on cemiplimab, one (17%) developed Grade 3 hepatitis that resolved with immunosuppression but led to drug discontinuation. No adverse events were observed in patients with MCC receiving pembrolizumab. One patient with BCC died 93 days after completion of radiation due to non-cancer and non-treatment related comorbidities. No unexpected systemic therapy related toxicities were identified in the available clinical documentation; however, complete toxicity assessment was limited by the retrospective design. Radiotherapy-specific acute and late toxicities were not systematically graded in the medical record and therefore could not be reported. Apart from Patient 12, whose initial upfront course of definitive radiation was stopped because of intolerance before systemic therapy was started, no interruption of consolidative radiation for toxicity was documented. 

## 4. Discussion

In three cohorts of seven patients with BCC, six patients with CSCC and two patients with MCC not amenable to upfront surgical resection, a 100% clinical CR by physical exam was observed in each group among patients who completed the induction systemic treatment and response-adapted consolidative radiation. Median DOOR was 26.2 months for BCC, 29.4 months for CSCC, and 27.1 months for MCC. Median treatment-free response was 18.1 months for BCC, 21.2 months for CSCC, and 20.4 months for MCC. Clinical responses were rapid after starting systemic therapy. Systemic therapy delivered before or concurrently with radiation was generally tolerable; one patient developed Grade 3 immune-mediated hepatitis on cemiplimab, which resolved with immunosuppression but required discontinuation. These results suggest that induction systemic therapy followed by response-adapted consolidative radiation is feasible in selected patients who are not appropriate for surgical resection; whether this approach is more effective or safer than radiation or systemic therapy alone cannot be determined from this uncontrolled series. 

Patients in this cohort did not pursue standard surgery due to large tumor size, unacceptable cosmetic morbidity, or non-surgical dermal lymphatic spread. This clinical conundrum is common: NMSC tends to occur on the head and neck in an aging, frail population where disfiguration or medical comorbidity may preclude surgery [21]. In response, there is a practical clinical need to establish an alternative treatment approach. While data from EMPOWER-CSCC-01 and other studies have suggested a 2-year treatment period in the definitive setting, prolonged systemic treatment often poses risk of immune-mediated toxicity as well as logistical and financial strain in an aging population [22]. Thus, consolidative radiation as a definitive treatment option was offered. The data from our cohort supports further exploration of one such approach, with no recurrences observed in any histologic subtype during follow-up after completion of radiation. 

While we report a 100% clinical CR by physical exam within each NMSC histological subtype, radiographic CR across the groups was lower at 33%. While one explanation for this discrepancy is that viable tumor persisted and tumor was not fully eradicated, the inaccuracy for radiologic imaging in NMSC was previously described [23]. Though our study inherently did not examine pathological response data in the absence of surgery, our data are reminiscent of the radiographic and pathological response rate discrepancy observed in cutaneous trials. Gross et al initially showed that while patients with CSCC treated with neoadjuvant cemiplimab had a pCR of 51%, 88% of those patients demonstrated PR or SD on imaging [10]. In a follow-up analysis, 12-month event-free survival was 89% and overall survival was 92% [14]. Additionally, the largest trial to date for patients with melanoma, another cutaneous tumor, treated with neoadjuvant pembrolizumab showed a pCR of 21% and radiographic CR of 6%, but event-free survival at 2 years was 72% [24]. These trials suggest that radiographic response is not consistently representative of outcome, perhaps because RECIST v1.1. may not be the most comprehensive tool to assess irregular, exophytic or ulcerated lesions on the skin surface. A follow-up study from the MATISSE trial suggests 18F[FDG]-PET/CT may be the modality to accurately identify response in CSCC [25]. Cutaneous malignancies are also unique in that surface lesions allow for clinical assessment of response. Further investigation with mapping biopsies in a future, larger trial is required to ensure full eradication of disease and also to determine whether clinical CR by examination can serve as a surrogate for pathologic response. Similarly, in the recently presented RAMPART trial, which prospectively enrolled 34 patients to receive neoadjuvant cemiplimab followed by definitive radiation, 18-month PFS was 86% and OS was 92%; however, the ORR by RECIST v1.1 was lower [26]. Larger-scale studies are needed to further clarify the discrepancy between radiologic, physical exam, and long-term outcome findings of cutaneous tumors treated in this manner. 

Neoadjuvant systemic treatment aimed to cytoreduce bulky tumors for surgical feasibility has robust and growing evidence in cutaneous malignancies [23]. Initial studies for vismodegib demonstrated reduction in size of resectable BCC tumors; later, case reports demonstrated more limited surgical resection in patients treated with neoadjuvant sonidegib [27,28]. A recent phase II trial of 12–14 weeks of induction vismodegib followed by seven weeks of concurrent vismodegib and RT in 24 patients with unresectable, locally advanced BCC resulted in response rates and 1-year locoregional control rates of 83% (confidence interval [CI], 59–96) and 91% (CI, 68–98), respectively; at 5 years, progression-free and overall survival rates were 78% and 83%. No grade ≥4 treatment-related adverse events occurred, and patients reported clinically meaningful benefit to quality-of-life based on a standardized questionnaire [29]. Meanwhile, neoadjuvant ICI is being increasingly used and investigated in resectable CSCC and MCC [10,16,23,30] and is now considered a standard of care for clinically detectable, measurable stage III melanoma on the basis of randomized trials [24,31]. Here, administration of induction systemic treatment objectively reduced tumor volumes, decreasing the amount of radiation delivered; additionally, the administration of ICI may target micro-metastatic disease and prime the immune system toward anti-tumoral immunity prior to consolidative radiation. Per more recent publication of the C-POST trial, there is also an indication to offer post-operative cemiplimab in CSCC, but further studies are needed to compare the neoadjuvant versus adjuvant approach [32].

Hedgehog inhibitor class toxicities warrant particular consideration in this population. In our cohort, dysgeusia affected 57% of patients receiving sonidegib and, together with nausea, led to dose reduction to alternate-day dosing in three patients—in some cases because of poor oral intake and failure to thrive. These findings are consistent with the long-term BOLT analysis, in which muscle spasms, alopecia, and dysgeusia were the most common adverse events and frequently led to dose interruption or discontinuation [4]. In elderly and frail patients, weight loss related to dysgeusia can be clinically significant. A defined, time-limited induction course followed by consolidative radiation, rather than continuous HHI therapy, may limit cumulative toxicity, but this hypothesis requires prospective evaluation that includes patient-reported outcomes.

Limitations of our study include a single-center, retrospective case series with small sample size. While all patients were discussed at a multidisciplinary tumor board, treatment was tailored to the specific comorbidities of each patient and did not follow a pre-specified rubric. The method of identifying patients may introduce selection bias: patients were identified retrospectively by the treatment they received rather than enrolled at the start of therapy, so patients who began systemic therapy but did not proceed to radiation were not captured, and an intention-to-treat analysis was not possible. The 100% clinical CR rate therefore describes outcomes among patients who completed both treatment components and should not be interpreted as the response rate of this approach in all patients who start induction therapy. The radiation doses delivered (55–70 Gy) are curative-intent on their own, and without a comparator the relative contributions of systemic therapy and radiation to disease control cannot be separated. DOOR includes time on treatment and is not a conventional duration-of-response endpoint, and follow-up after completion of radiation remains limited, with few patients followed beyond 2 years; with small subgroups and no recurrence events, time-to-event analyses were not informative. Two patients (Patients 8 and 12) deviated from the planned treatment sequence; results excluding them are provided as a sensitivity analysis. Finally, ctDNA-guided timing of radiation in MCC was applied in only two patients and is exploratory. Similarly, while the intent of this approach is to employ radiation as a definitive, limited treatment, we were limited in obtaining radiation-specific toxicities and quality-of-life, functional and cosmetic outcomes. All conclusions drawn from this small cohort of highly selected patients are meant to be hypothesis-generating for further prospective, comparative investigation. Additional areas worthy of future investigation include clinical or radiographic biomarkers of pathologic response and de-escalation of radiation in a response-adapted fashion. 

## 5. Conclusions

Resection of locally advanced NMSC can yield undesired cosmetic deficits and confer significant surgical morbidity, particularly in an aging population. Therefore, there is an unmet clinical need to develop less invasive treatment algorithms for such cases. Our data suggest that induction systemic therapy followed by response-adapted consolidative radiation is feasible and may be associated with rapid clinical responses and durable disease control in highly selected patients. These findings are hypothesis-generating. Future prospective trials are needed to refine patient selection and sequencing of therapy for this approach. 

## Figures and Tables

**Table 1:** Baseline characteristics, treatment, and response.

Patient Demographics	BCC, n = 7	SCC, n = 6	MCC, n = 2	Total, n = 15
Age, years, median [range]	72 [63–78]	74 [54–78]	62 [46–78]	73 [46–78]
Follow-up length, months, median [range]	27.6 [10.6–37.9]	30.1 [8.2–55.6]	27.9 [21.9–33.8]	28.2 [8.2–55.6]
Gender, count (%)				
Female Male	2 (29)	0 (0)	0 (0)	2 (13)
	5 (71)	6 (100)	2 (100)	13 (87)
Tumor Location, count (%)				
Face	3 (43)	2 (33)	1 (50)	6 (40)
Ear	2 (29)	2 (33)	0 (0)	4 (27)
Scalp	1 (14)	1 (17)	0 (0)	2 (13)
Upper Extremity	0 (0)	1 (17)	1 (50)	2 (13)
Neck	1 (14)	0 (0)	0 (0)	1 (7)
Reason Surgery Deferred, count (%)				
Tumor Size	5 (71)	3 (50)	2(100)	10 (67)
Cosmetic Morbidity	2 (29)	1 (17)	0 (0)	3 (20)
Dermal Lymphatic Spread	0 (0)	2 (33)	0 (0)	2 (13)
Treatment Regimens	BCC, n = 7	SCC, n = 6	MCC, n = 2	Total, n = 15
Systemic Therapy, count (%)				
Sonidegib	7 (100)			7 (47)
Cemiplimab		6(100)		6 (40)
Pembrolizumab			2 (100)	2 (13)
Duration of Systemic Therapy, months, median [range]	6.4 [1.1–15.3]	11.1 [4.8–18.8]	7.5 [7.4–7.6]	7.6 [1.1–18.8]
Treatment Response	BCC, n = 7	SCC, n = 6	MCC, n = 2	Total, n = 15
Time to Clinical Response, days, median [range]	40 [25–45]	21.5 [20–38]	22 [22]	25 [20–45]
Clinical Response, count (%)				
Complete Response by exam	7 (100)	6 (100)	2 (100)	15 (100)
Radiographic Response, count (%)				
Complete Response	3 (43)	2 (33)	0 (0)	5 (33)
Partial Response	1 (14)	2 (33)	0 (0)	3 (20)
Stable Disease	2 (29)	2 (33)	2 (100)	6 (40)
Not available	1 (14)	0 (0)	0 (0)	1 (7)
DOOR, months, median [range] Treatment Free Interval, months, median [range]	26.2 [9.8–36.4] 18.1 [2.0–22.5]	29.4 [7.5–54.9] 21.2 [3.4–37.5]	27.1 [21.2–33.1] 20.4 [14.5–26.3]	26.9 [7.5–54.9] 20.5 [2.0–37.5]

## Data Availability

Research data are stored in an institutional repository and will be shared upon request to the corresponding author.

## Supplementary Materials

The following supporting information can be downloaded at: https://www.mdpi.com/article/10.3390/curroncol33100602/s1, Table S1: Additional Baseline and Treatment Data.

## Abbreviations

The following abbreviations are used in this manuscript:

NMSC	Non-melanoma skin cancer
CSCC	Cutaneous squamous cell carcinoma
BCC	Basal cell carcinoma
MCC	Merkel cell carcinoma
