# Supplementary material for: Induction Systemic Therapy Followed by Response-Adapted Consolidative Radiation in Locally Advanced Non-Melanoma Skin Cancer: A Retrospective Case Series
Source: Curr Oncol. 2026 Oct 6;33(10):602. doi: 10.3390/curroncol33100602 (PMC13703689; doi:10.3390/curroncol33100602)
Supplement: Supplementary file 1 [file curroncol-33-00602-s001.zip › curroncol-4419595-supplementary.pdf]

Patient ID	Age at Treatment Onset	Comorbidities	ECOG	Histology	Clinical Stage (if primary) or Recurrence	Disease Location	Reason Surgery Not Pursued	Systemic Drug and Dose	Duration of Systemic Therapy (Months)	Radiation Dose (Gy)	Radiation Fractions	Clinical Response	Radiographic Response	ctDNA Response	Duration of Observed Response (Months)	Survival Status at Last Follow up	Toxicity (Grade)
**1**	46	HIV on therapy, chronic HBV, treated HCV, smoking	0	MCC	T3N0M0	Upper Extremity	Morbidity/Cosmesis: Extensive reconstruction required	Pembrolizumab 200mg	2.8	56	28	Complete Response	Stable Disease	Clearance	21.2	Alive	None
**2**	78	Coronary artery disease, atrial fibrillation	1	MCC	T3N0M0	Face	Morbidity/Cosmesis: Extensive reconstruction required	Pembrolizumab 200mg	7.6	60	30	Complete Response	Stable Disease	Clearance	33.1	Alive	None
**3**	72	Coronary artery disease	1	CSCC	T3N3bM0	Neck	Morbidity/Cosmesis: Extensive reconstruction required	Pembrolizumab 200mg	4.8	50	22	Complete Response	Partial Response	N/A	7.5	Alive	None
**4**	78	Emphysema, peripheral arterial disease	2	CSCC	Recurrent, node positive	Ear	Technically Unresectable: Dermal lymphatic spread	Cemiplimab 350mg	4.9	60	30	Complete Response	Partial Response	N/A	27.7	Alive	Hepatitis (G3)
**5**	75	Congestive heart failure	1	CSCC	T3N0M0	Ear	Morbidity/Cosmesis: Total auriculectomy required	Cemiplimab 350mg	10.4	70	35	Complete Response	Stable Disease	N/A	31.1	Alive	None
**6**	54	Treated HCV	1	CSCC	T3N2bM0	Upper Extremity	Morbidity/Cosmesis: Extensive reconstruction required	Cemiplimab 350mg	11.8	60	30	Complete Response	Complete Response	N/A	18.4	Alive	None
**7**	76	Coronary artery disease, type 2 DM	1	CSCC	T4N0M0	Scalp	Technically Unresectable: Sagittal sinus invasion	Cemiplimab 350mg	13.4	60	30	Complete Response	Stable Disease	N/A	33.7	Alive	None
**8**	75	Rheumatoid arthritis on tofactinib	1	CSCC	Recurrent, node negative	Face	Technically Unresectable: Dermal lymphatic spread	Cemiplimab 350mg	18.8	70	35	Complete Response	Complete Response	N/A	54.9	Alive	None
**9**	63	Recent stroke on dual antiplatelet	2	BCC	T3N0M0	Ear	Morbidity/Cosmesis: Total auriculectomy required	Sonidegib 200mg	1.1	60	30	Complete Response	N/A	N/A	11.7	Alive	Fatigue (G1)
**10**	72	Type 2 DM (poorly controlled), treated HCV	1	BCC	Recurrent, node negative	Scalp	Morbidity/Cosmesis: Mastoid invasion	Sonidegib 200mg	8.6	60	30	Complete Response	Partial Response	N/A	9.8	Expired	None
**11**	68	Emphysema, type 2 DM	1	BCC	Recurrent, node negative	Ear	Morbidity/Cosmesis: Total auriculectomy required	Sonidegib 200mg*	5.7	60	30	Complete Response	Complete Response	N/A	15.4	Alive	Nausea (G3)
**12**	76	Stage 3B chronic kidney disease	3	BCC	T3N0M0	Scalp	Medically unresectable: Age, comorbidities, performance status	Sonidegib 200mg*	3.4	40	16	Complete Response	Stable Disease	N/A	26.9	Alive	Dysgeusia (G2)
**13**	72	Type 2 DM, gout	1	BCC	T3N0M0	Ear	Medically Unresectable: Age, comorbidities	Sonidegib 200mg	6.4	66	33	Complete Response	Complete Response	N/A	26.2	Alive	Dysgeusia (G1)
**14**	78	Type 2 DM (poorly controlled), indwelling urinary catheter	3	BCC	T4N0M0	Scalp	Morbidity/Cosmesis: Extensive reconstruction required	Sonidegib 200mg*	9.0	55	20	Complete Response	Complete Response	N/A	28.0	Alive	Dysgeusia (G2)
**15**	74	Congestive heart failure, atrial fibrillation	1	BCC	T3N0M0	Nose	Morbidity/Cosmesis: Extensive reconstruction required	Sonidegib 200mg	15.3	60	24	Complete Response	Stable Disease	N/A	36.4	Alive	None
